# Compatibilizing of cotton fabric with hydrophobic drug cover layer for anti-inflammatory performance with the implementation of ibuprofen

**DOI:** 10.1038/s41598-024-57883-5

**Published:** 2024-03-27

**Authors:** Aneta Kopańska, Marek Brzeziński, Weronika Gonciarz, Zbigniew Draczyński

**Affiliations:** 1 Institute of Material Science of Textiles and Polymer Composites, Lodz University of Technology, Żeromskiego 116, 90-543 Lodz, Poland; 2 Centre of Molecular and Macromolecular Studies, Polish Academy of Sciences, Sienkiewicza 112, 90-363 Lodz, Poland; 3 Department of Immunology and Infectious Biology, Faculty of Biology and Environmental Protection, University of Lodz, Banacha 12/16, 90-237 Lodz, Poland

**Keywords:** Health care, Medical research, Biomedical engineering, Surface chemistry

## Abstract

This paper presents active analgesic and anti-inflammatory dressings based on cotton woven material with surface functionalization enabling drug implementation. For this purpose, lactide was polymerized on the surface of cotton textiles to achieve better compatibility with hydrophobic drug and polylactide (PLA)-based macromolecules. Subsequently, ibuprofen-loaded PLA and PLA-PEG were implemented through the exhausting method. Such material was tested for cytotoxicity (toward L929 mouse fibroblasts) and anti-inflammatory activity (towards human Hs68 fibroblasts) based on the secretion of pro-inflammatory cytokines IL-1β and TNF-α. The results showed that the drug attachment and its performance are influenced by a combination of mercerization, bleaching and polylactide grafting, and the release of ibuprofen depends on the drug-loaded layer structure. Moreover, we show that cotton woven fabric with ibuprofen-loaded PLA and PLA-PEG cover layers had anti-inflammatory properties. These new dressings may open possibilities for developing prolonged analgesic and anti-inflammatory materials for wound healing or transdermal drug delivery.

## Introduction

Cotton dressings, or looking more broadly, cellulosic materials, have been a source of scientific interest for years and still have a strong presence in professional health care^1^. Nowadays, the key issue is to elaborate on a method of improving the properties of the dressing, which was initially passive, to active (or so-called smart) protection from external conditions, and promoting tissue regeneration by creating an advantageous healing environment^2^. This includes not only creating new materials but also modifying classical cotton or cotton/viscose materials, which are widely available on the market^3–5^. However, the latest trends focus more on cellulosic nanomaterials as potentially effective drug carriers and general wound healing equipment^6^.

Ibuprofen (IBU) belongs to the group of non-steroidal anti-inflammatory drugs (NSAIDs) and is used to relieve pain, fever and inflammation. It inhibits the production of prostaglandins by reducing the activity of the cyclooxygenase enzyme in constitutive (COX-1) and induced (COX-2) forms^7^. However, an oral application may be associated with some unfavorable side effects such as gastrointestinal (GI) complications^8^; therefore, alternative ways for IBU delivery are being investigated, and topical applications are among them^9^. Some solutions for dressings have already been commercialized, for example, Biatain^®^ Ibu Non-adhesive or Biatain^®^ Ibu Soft-Hold (Coloplast A/S, Denmark), a polyurethane foam dressing containing 0.5 mg ibuprofen on 1 cm^2^. The wound’s exudate, which the foam gradually absorbs, influences the release of the drug from the dressing^10^. Pain reduction was observed during in vivo studies on patients with various types of wounds^11–14^.

Besides polyurethane foam, other polymeric materials, like PLGA or collagen, were examined to incorporate ibuprofen for wound healing. They were formed in films^15^, hydrogels^16,17^, or membranes^18^. In turn, when accompanying polylactide to create active dressings, IBU is often applied as an addition to spinning solution for electrospun fibers, which then are converted into mats, membranes and scaffolds^19–21^. In the case of cellulose and its derivatives, an example is cellulose nanofibrils, which were grafted with ibuprofen through esterification. This drug delivery system achieved efficiency of 151.38 mg/g of loaded ibuprofen^22^. Another one is bacterial cellulose acting as a carrier of IBU esters, l-valine isopropyl ester ibuprofenate and l-leucine isopropyl ester ibuprofenate^23^. On the other hand, TEMPO-oxidized cellulose was formed into hydrogel with the addition of Ca^2+^ ions, and then free ibuprofen or one complexed with β-cyclodextrin was incorporated^24^.

This paper presents active analgesic and anti-inflammatory dressings based on cotton woven material with surface functionalization enabling drug implementation. For this purpose, lactide was polymerized on the surface of cotton textile to achieve better compatibility with a drug-loaded polylactide cover layer. Ring-opening polymerization (ROP) is a well-known method of polymerization of lactide^25–27^. The synthesis of PLA can be performed on the surface of cellulose fibers, where the hydroxyl groups of cellulose molecules are acting as an initiator^28^. Two PLA forms were chosen to enclose ibuprofen, star-shaped PLA, and PLA-PEG copolymer, which have different affinity for water, and therefore, to hydrophilic cotton fibers. They were intended to cover modified cotton fabric by exhausting method and act as the top, active layer in drug delivery. The kinetics of drug release from both drug carriers were compared. Subsequently, the new dressings were tested for cytotoxicity and anti-inflammatory effects.

## Materials and methods

All used reagents, unless otherwise noted, were purchased from Merck KGaA, (Darmstadt, Germany). Raw cotton fabric (1/1 plain weave, thickness 0.33 mm, surface mass 120 g/m^2^, yarn density 18 tex (which means 18 g/1,000 m), yarn diameter 0.17 mm) was kindly provided by PTB (Pabianice, Poland). l,l-LA was purchased from Purac (99%, The Netherlands), recrystallized from dry 2-propanol and stored under reduced pressure. Trifluoromethanesulfonic acid (98%), methyl-β-cyclodextrin (MβCD, 99%, methylation degree per glucose unit: 1.6–2.0) was used without further purification. Tin(II) octoate (2-ethylhexanoate, Sn(Oct)2, Aldrich, 95%) was purified by two high-vacuum distillations at 140 °C and 3 × 10^−1^ mbar and stored under vacuum. 1,2-dichloromethane (DCM) was distilled over P_2_O_5_ and stored under reduced pressure. Tetrahyfrofurane (THF, analytical grade) was distilled over sodium pellets and stored under vacuum. Methanol (analytical grade) was used as received.

**Figure 1:**
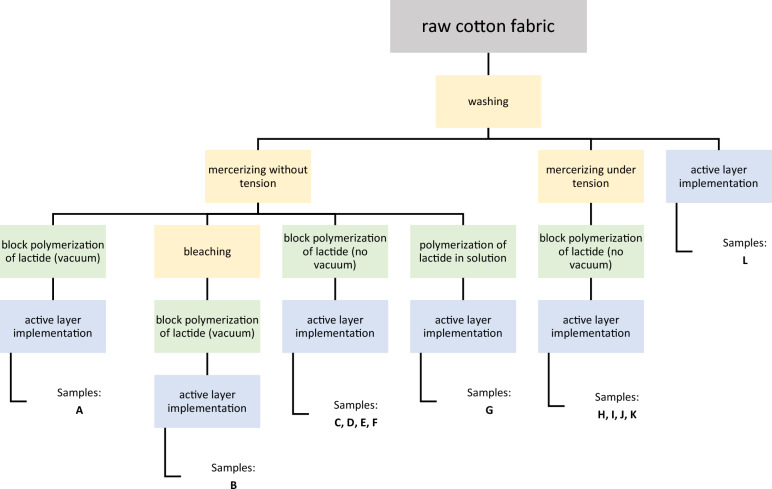
General scheme of different preparations paths for cotton functionalising. Orange presents the pretreatment stage, green polymerization of lactide, and blue—implementation of ibuprofen.

The main goal of the experiment was to investigate the influence of base material (cotton fabric) preparation on drug performance and its efficiency. Therefore, a wide range of samples was made, with different variants of the preparation process, as shown in Fig. 1. Before the polymerization stage, a raw cotton fabric was subjected to few pretreatments which included washing, bleaching, and mercerizing. After that, l,l-lactide was polymerized in three different ways, and then the samples were impregnated with an active layer by the exhausting method, which was widely discussed by Bonet^29^. Details of every stage are described in the following paragraphs, including chemicals used and modification routine. In the most extensive version of the process, a cotton sample was washed, bleached, mercerized, modified by lactide and impregnated with a carrier containing ibuprofen (sample B). In the shortest version, one of the samples (sample L) was only washed and impregnated with IBU dispersion (Table 1).

**Table 1:** Detailed schedule of modification steps for cotton samples.

Sample	Pretreatment	Polymerization	Exhausting method/polymer chosen for the top layer
A	Washing, mercerization (no tension, 20 min)	Bulk (vacuum)	1-step (star PLA)
B	Washing, mercerization (no tension, 20 min), bleaching	Bulk (vacuum)	1-step (PLA-PEG)
C	Washing, mercerization (no tension, 3 min),	Bulk (no vacuum)	1-step (PLA-PEG)
D	Washing, mercerization (no tension, 20 min),	Bulk (no vacuum)	1-step (PLA-PEG)
E	Washing, mercerization (no tension, 3 min)	Bulk (no vacuum)	1-step (star PLA)
F	Washing, mercerization (no tension, 20 min)	Bulk (no vacuum)	1-step (star PLA)
G	Washing, mercerization (no tension, 20 min)	Solution	1-step (star-PLA)
H	Washing, mercerization (under tension, 3 min)	Bulk (no vacuum)	1-step (PLA-PEG)
I	Washing, mercerization (under tension, 20 min)	Bulk (no vacuum)	1-step (PLA-PEG)
J	Washing, mercerization (under tension, 3 min)	Bulk (no vacuum)	1-step (star PLA)
K	Washing, mercerization (under tension, 20 min)	Bulk (no vacuum)	1-step (star PLA)
L	Washing	No	2-step (PLA-PEG + star PLA)
Raw (reference sample)	Washing	No	No

### Characteristics of the cover layers containing IBU

Star-shaped polymers are characterized by a single branch point from which linear chains emerge. The functionality of this point is referred to as the number of arms leaving it (linear chains). Star-shaped polymers can be prepared with a convergent (“arm-first”, “arm-in”), divergent (“core-first”, “arm-out”), or grafting-onto approach^30^. Various star-shaped PLA structures can be prepared as summarized by Michalski et al.^31^. For this research, star-shaped PLLA was synthesized via ring opening polymerization method, with methylated β-cyclodextrin as an initiator and stannous octoate (Sn(Oct)_2_) as a catalyst, as described in the literature^32–34^. Briefly, the bulk polymerization was carried out under reduced pressure in a sealed glass at 130 °C. The Sn(Oct)2 (10 mg, 0.025 mmol) was used as a catalyst and MnβCD (0.46 mmol, 0.602 g) was used as initiators l,l-lactide (35.7 mmol, 5.15 g) polymerization. After polymerization, the prepared polymers (star-PLA was dissolved in DCM and purified by slow precipitation to cold methanol and dried for 24 h under reduced pressure. star-PLA possesses 12 wt.% of MnβCD in the polymer structure.

PEG-PLA copolymers are already known as a matrix for the preparation of drug delivery systems^35–38^. Some in vivo examinations were also conducted^39,40^. Moreover, the important feature of PLA-PEG copolymers is the increased surface wettability of materials made of them^41^. For the purposes of ibuprofen implementation, the PLA-PEG copolymer was synthesized according to the procedures described by Michalski et al.^42^. l,l-lactide (5.70 g, 40 mmol) was polymerized in a glass ampule under reduced pressure in tetrahydrofuran (THF) at 80A °C with stannous octoate Sn(Oct)_2_ as a catalyst (1 mL of a 0.25 mol L^−1^ solution in dry THF) and at the presence of mPEG-OH (0.25 g, 1.25 mmol) as an initiator^42^. Subsequently, after 24 h, PLA-PEG copolymers was precipitated to cold methanol and dried under vacuum.

### Cotton pretreatment

#### Washing

The cotton fabric was washed in anionic detergent Roksol M7 (PCC Exol SA, Brzeg Dolny, Poland) with a ratio of 10 g of detergent to 1 L of water for 1 h. The temperature of the bath was 60 °C. Then, the material was rinsed with cold water, squeezed lightly, and left to dry freely at room temperature.

#### Mercerizing

Mercerizing is a process of modifying cotton fibers structure with sodium hydroxide solution, which results in some physical changes such as increased strength, water affinity, as well as, chemical reactivity^43^. In this case, mercerization allowed for the multiplication of active centers on the surface of cotton fabric, so the following polymerization of lactide would have an easier access to initiating OH groups. For this purpose, a 17% sodium hydroxide solution was prepared with the addition of Roksol M7 as a wetting agent with a ratio of 2 g to 1 L of solution. The modification was conducted at temperature of 21 °C, for 3 or 20 min. Some samples were also put under tension during the process (as seen in Table 1). After the set time passed, samples were neutralized in 3% acetic acid, rinsed, and dried for 24 h at room temperature and 60–65% relative humidity level.

#### Bleaching

According to Hausner^43^, the following recipe for bleaching was provided: samples were put in a heated up to 80 °C bath containing 30% hydrogen peroxide (H_2_O_2_, with a ratio of 30 mL/L), Roksol M7 (2 g/L), NaOH (1.5 g/L), and sodium silicate (8 g/L). After 1 h of bathing, samples were rinsed with cold water and left for drying for 24 h at room temperature and 60–65% relative humidity level.

#### Lactide polymerization on the cotton surface

To decrease the hydrophilic properties of cotton fabric surface and increase compatibility with enriched with IBU polylactide layer, grafting of l,l-lactide was performed in three different conditions, bulk polymerization with (1) or without the use of vacuum (2) with stannous (II) octoate Sn(Oct)_2_ as catalyst, and cationic polymerization (3) with triflic acid (trifluoromethanesulfonic acid, CF_3_SO_3_H). Before starting synthesis, l,l-lactide was crystallized from dry 2-propanol, and sublimed. Formerly pretreated (washed, mercerized, bleached) cotton samples were conditioned for 24 h at 130 °C to remove excessive moisture.

##### Approach 1

 5 g of l,l-lactide and two cotton samples 2 × 2 cm were placed in the glass ampule and dried under reduced pressure within 3 h. After that, 10 mg of Sn(Oct)_2_ was added, and the glass vessel was sealed with a burner under a high vacuum. The ampule was kept in oven at 130 °C within 24 h until newly formed polymer was in solid state.

##### Approach 2

The 5 × 5 cm samples were immersed in a 20 mL solution of distilled dichloromethane (CH_2_Cl_2_) containing 25 mg of Sn(Oct)_2_ under an argon atmosphere and mixed at room temperature for 0.5 h. Then, dichloromethane was removed, and samples were dried in a vacuum for 1 h. The prepared sample of 5 g of l,l-lactide was thoroughly spread out to obtain a thin layer, which would gradually penetrate the structure of the fabric during melting. For this variant, during polymerization vacuum was not used and there were no changes to other conditions (130 °C and 24 h of time).

##### Approach 3

Two samples of cotton (2 × 2 cm) and 5 g (0.035 mol) of l,l-lactide were placed in a Schlenk flask. The flask was degassed under reduced pressure, filled with argon, and sealed with a silicone septum. Next, 10 mL of dried CH_2_Cl_2_ was added. After the complete dissolution of the lactide, 50 µL of triflic acid was introduced through the septum, and the polymerization was conducted at room temperature for 24 h. At the end of every procedure, samples were rinsed twice with dichloromethane to remove unbonded polymer, which was then precipitated into methanol for further examinations. In contrast, the samples dried freely at room temperature without additional purification.

#### Covering with the active layer

Ibuprofen was purchased from Pol-Aura (Morąg, Poland). In a 1-step active layer application, 0.2 g of IBU and 0.4 g of star-PLA were added to 100 mL of dichloromethane and stirred for 15 min to prepare the drug carrier solution. Then, the samples with a total mass of 2.2 g were put into the bath and stirred for 24 h at room temperature. The last 15 min, the temperature was raised to 50 °C. The same procedure was specified for the PLA-PEG. The ratio of polymer to IBU amount was again kept at 2:1. Whereas the 2-step application included firstly stirring the samples in 150 mL of dichloromethane with 0.2 g PLA-PEG at room temperature and then adding 0.2 g of IBU and 0.2 g of star-PLA dissolved simultaneously in 50 mL of dichloromethane and stirring for another 24 h. The last 15 min ran at the temperature of 50 °C, just as in the 1-step method. All samples were dried flat afterwards without any rinsing. The theoretical amount of loaded ibuprofen results from the proportion of the drug in correlation to the amount of cotton fabric used and is 1 mg/cm^2^.

#### Characterization of the modified cotton

The wetting test of cotton was performed to demonstrate the degree of hydrophobization of the cotton fabric surface after the polymerization of lactide. In this research, the static water contact angle was determined using the sessile drop method. Measurements were performed using the DSA100 goniometer by Kruss (Germany). The built-in camera photographed the droplet positioned on the surface of the fabric after 1 s, and the angle was determined and calculated automatically. The measurement done for every sample had 4 repetitions. Then, the average of the measurements and standard deviation were calculated. Fourier-transform infrared spectroscopy (ATR-FTIR) spectra were measured with a Nicolet 6700 spectrometer to observe structural changes on the surface of cotton. The examination was obtained by adding 64 scans at a 2 cm^−1^ resolution. The molecular weight of the synthesized PLA was determined by size-exclusion chromatography (SEC) using an Agilent Pump 1100 Series with 2× PLGel 5 microns MIXED-C columns and methylene chloride as an eluent.

To determine the release of ibuprofen from the dressings, 1 × 1 cm samples were cut and put into 20 mL of phosphate buffered saline (PBS 0.1 M, pH 7.4) at room temperature. The release profile of IBU in PBS was examined by recording UV–VIS spectra at 220 nm using the spectrophotometer Thermo Scientific™ Evolution 220 (USA) with Insight software. Samples were measured triplicate for every point, and the calculations were made to determine the average and standard deviation [SD] of the measurements. The calibration curve was prepared with R^2^ coefficient of 0.999, and the wavelength for the drug concentration measurements was 220 nm.

### In vitro examinations

#### Cell culture

Reference L929 mouse fibroblasts (purchased in LGC Standards, Middlesex, UK) and human Hs68 skin fibroblasts (CRL-1635™, purchased in American Type Cell Cultures (ATCC), Rockville, MD, USA). Mouse L929 fibroblasts were cultured at 37 °C in a 5% CO_2_ in Roswell Park Memorial Institute (RPMI)-1640 medium supplemented with 10% heat-inactivated fetal bovine serum (FBS) and standard antibiotics: penicillin (100 U/mL) and streptomycin (100 µg/mL), while the Hs68 cell line was grown in high glucose RPMI-1640 medium containing 10% FBS, 100 U/mL penicillin, and 100 µg/mL streptomycin (all cell culture components were from Biowest, Nuaillé, France). Cell cultures were supplemented with fresh medium two or three times weekly to keep them in the log phase. The confluent monolayer was treated with 0.25% trypsin–EDTA solution (Biowest, Nuaillé, France) to passage.

#### Direct contact cytotoxicity

Direct contact cytotoxicity assay of the tested materials, row cotton and A-L was examined using L929 and Hs68 cell lines (density of 2 × 10^5^ cells/mL) according to the ISO norm 10993-5 (International Organization for Standardization, 2009; Biological evaluation of medical devices Part 5: tests for in vitro cytotoxicity), with a use of the 3-(4,5-dimethylthiazol-2-yl)-2,5-diphenyltetrazolium bromide (MTT) reduction assay, as previously described^44^. Under aseptic conditions, each tested material was placed in an individual well of the non-adherent Nunclon Delta Surface 24-well culture plate (Nunc, Thermo Fisher Scientific, Waltham, MA, USA) in four replicates each. During the 24 h incubation in 1 mL of culture medium at 34 °C and 5% CO_2_, the tested materials absorbed the liquid. Next, the tested materials, row cotton and A-L samples, were cut into small pieces and transferred to a 96-well cell culture plate containing previously prepared L929 and Hs68 cells. The cell cultures in medium alone, without the tested materials, were used as a positive control (PC) for cell viability, whereas cells treated with 0.03% H_2_O_2_ were used as a negative control (NC), i.e. 100% dead cells due to lysis, and additionally as a control—raw (unmodified) cotton. The absorbance was measured spectrophotometrically using a Multiskan EX plate reader (Thermo Scientific, Waltham, MA, USA) at 570 nm. MTT reduction was related to untreated cells (%) = (absorbance of treated cells/absorbance of untreated cells × 100%) × 100%.

### Anti-inflammatory properties of tested materials—extracellular secretion of anti-inflammatory cytokines

To assess the inflammatory effect of the tested materials, raw cotton and A-L samples, the level of anti-inflammatory cytokines (IL-1β and IL-8) was evaluated. The Hs68 cells were cultured for 24 h with tested materials alone and with tested materials in combination with lipopolysaccharide (LPS) from *E. coli* (1 µg/mL) to assess the anti-inflammatory potential of extracts. The inflammatory process in cell lines was induced by the treatment with LPS from *E. coli* (1 µg/mL), while according to Batool (2018), ibuprofen alone (50 µg/mL) as well as in combination with LPS from *E. coli* (1 µg/mL) was used as a control of anti-inflammatory action. The level of cytokines IL-1β, and IL-8 concentration in cell culture supernatants was tested using commercial ELISA (Invivogen Houston, TX, USA) which were performed according to the manufacturer protocol (Invivogen Houston, TX, USA). The test's sensitivity was 1.5 pg/mL for IL-B and 1.7 pg/mL for TNF α.

### Statistical analyses

Data are presented as mean values ± standard deviation (SD). The differences between tested variables were assessed using Statistica 13 PL software (https://statistica.software.informer.com/13.3software, Krakow, Poland) with a nonparametric Mann–Whitney U test (statistical comparison among two groups) or the Kruskal–Wallis test (statistical comparison among different groups). The results were considered statistically significant when p < 0.05.

## Results and discussion

Different affinity for water is a known issue in the adhesion between polymeric materials, which is solved by surface modifications of the components and the use of compatibilizers or coupling agents. In this study, the targeted use of the produced cotton-polylactide active material eliminated procedures and reagents that do not meet the safety requirements for potential patients, so the utilization of chemicals was limited to the necessary minimum. Therefore, the grafting of PLA macromolecules on the hydrophilic cotton surface was performed by ring-opening polymerization of lactide and stannous octoate as a catalyst.

Hydroxyl groups from cellulose macromolecules were used as the initiator of lactide polymerization, so the first goal was to make them more accessible for synthesis. This was achieved by mercerization, which converts a crystalline form of cellulose I to cellulose II, resulting in greater chemical reactivity of the cotton surface. Moreover, mercerizing removed some impurities from the fabric, as well as bleaching, which was conducted in some cases. All other auxiliaries and solvents were supposed to be washed out carefully or evaporated from the resulting material; therefore, in the end, the dressing consisted of three components: cellulose, polylactide or PLA-PEG copolymer and ibuprofen, where cellulose in the form of cotton fabric formed a strengthening base, PLA acted as a compatibilizer and drug carrier, while IBU was responsible for anti-inflammatory functions.

### Results of cotton base modifications

A major part of this study was creating a polylactide layer, which would be an intermediary between cellulose and IBU/PLA top layer and bond them sufficiently enough to ensure the long-acting effect of the dressings. For that reason, three methods of grafting polymerization were chosen, and among them one was bulk polymerization in atmospheric conditions. This kind of synthesis is not a common technique because of the difficulty of removing water and oxygen, thus reaching high levels of PLA molecular weight^25^. However, when the molecular weight is not an issue, it might still be attractive for economic reasons and relative ease of execution.

The relationship between the polymerization conditions and the length of the polylactide macromolecule chain can be confirmed by analyzing the molecular weight of the polymer obtained in each technique (bulk polymerization in or without vacuum and cationic one in solution, Table 2). The decrease of this value is significant in the case of atmospheric polymerization, and it can be correlated to an increased number of initiating centers from environmental moisture. Moreover, the organoleptic examination proved the unsolid, viscous character of the obtained layer and its inability to solidify, which is more characteristic of an oligomeric PLA. On the other hand, the molecular weight had the lowest polydispersity, mainly 1.2 (1.3), except for the material rinsed from cotton mercerized under tension in 3 min (samples H, J, polydispersity 1.7). The largest weight dispersion was observed in the case of polymerization in vacuum (samples A and B) in parallel with the largest chain lengths; however, in this case, previous bleaching of cotton lowered all these values, as seen in sample B. The high molecular weight distribution of PLA in this approach could be caused by the different reactivity of the hydroxyl groups of cellulose available on the surface^45^, as the reactivity of the initiator influences propagating processes^46^. Some reports indicate water as an ingredient supporting the control of chemical accessibility of selected hydroxyl groups^47^, but in this case the reaction was to take place in an anhydrous environment to obtain high quality polymer. As one can see, under identical conditions, bleaching can help to uniform the weight distribution, but also reduces the average molecular weight. It is worth mentioning that the same results (lower weight distribution, lower *M*_*w*_) occur after polymerization without vacuum (samples I-L).

**Table 2:** The molecular weight of PLA synthesized in various methods.

Sample	Polymerization	*M*_w_ (Weight av. molecular weight)	*M*_p_ (molecular weight of the highest peak)	Polydispersity
A	Bulk polymerization in vacuum	142,000	77,000	3.1
B	Bulk polymerization in vacuum	84,000	52,000	2.1
G	Cationic polymerization in solution	27,000	27,000	1.8
I, K	Bulk polymerization, no vacuum	1700	1200	1.2
F, D	Bulk polymerization, no vacuum	1700	1300	1.3
H, J	Bulk polymerization, no vacuum	2900	2600	1.7
E, C	Bulk polymerization, no vacuum	1500	1300	1.2
L	Bulk polymerization, no vacuum	1500	1300	1.2

**Figure 2:**
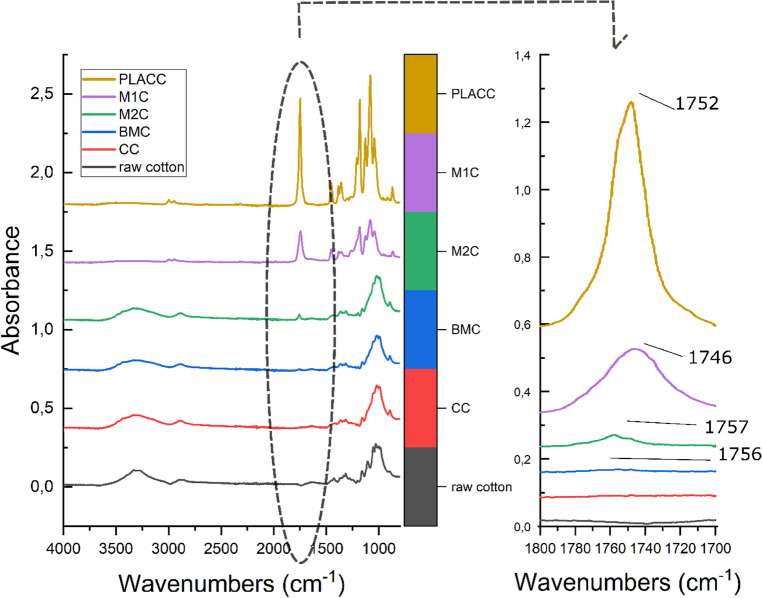
IR spectra of synthetized PLA material and functionalized cotton. In order from the top: (PLACC) PLA from cationic polymerization, (M1C) mercerized cotton with grafted PLA, (M2C) mercerized cotton after rinsing out of unbound PLA, (BMC) mercerized and bleached cotton with grafted PLA after rinsing out of unbounded PLA, (CC) cotton with PLA synthetized via cationic polymerization and rinsed twice, and raw cotton as a reference.

As IR spectra revealed, a small amount of PLA formed and bonded with the cotton surface. In the case of bulk polymerization, when comparing an image of mercerized cotton with PLA before and after rinsing from excessive polymer, proportions of PLA and cellulose nearly change to the opposite (M1C and M1C, Fig. 2). However, the presence of PLA is visible due to the peak at 1746 cm^−1^ (M1C) and 1757 cm^−1^ (M2C). Bands in this area show vibration of C=O of aliphatic ester^48^. The intensity of this peak is even lower in the case of cotton, which was previously mercerized and bleached and then modified by polymerization of lactide in a vacuum (BMC, 1756 cm^−1^). The poorest results were achieved after cationic polymerization, where after rinsing, no PLA was detected on the spectra, which showed a view of mercerized cotton. Additionally, after rinsing the nonwoven fabric, the solution contained significant amounts of polylactide (PLACC). Other changes, including the presence of the monomer, are mostly unrecognizable and difficult to clearly define due to the strong influence of cellulose.

The obtained results showed interesting changes in static contact angle measurement after polymerization (Table 3). The boundary value between the hydrophilic and hydrophobic properties of the material is 90°. Angles exceeding this value indicate a hydrophobic material, and the larger the angle, the greater the hydrophobicity of the sample appears. Similarly, for values below 90°, the lower the value, the greater the hydrophilicity of the material, to the point where angle measurement is impossible due to liquid absorption by the cotton. This examination with its limitations^49^ served as an auxiliary information of cotton compatibilization with polylactide layers. A high hydrophobization level of cotton fabric was achieved for samples A, B, and G, and a strong correlation can be seen between contact angle values and molecular weight (*M*_w_) of grafted PLA. It was shown that high grafting degree of polymers on the surface of cellulose may lead to the maximum contact angle of 134. 2°^50^. On the other hand, low molecular PLA showed varied effects of hydrophobization, from negligible changes (when a drop of water immediately soaks into the fabric, as in raw cotton) to values slightly exceeding 90°, ending with 100° in the case of cotton mercerized under tension and soaking of the droplet after a few seconds. It appeared that the hydrophilic properties of the cotton fabric surface outweighed the hydrophobic polylactide. After the drug carrier loaded with IBU was implemented, such characteristics of the sample surfaces remained mostly the same or the hydrophobicity only slightly increased, except samples C, D, and F. The main difference was in the behavior of samples of cotton mercerized without tension and regardless of mercerization time. The only sample that remained hydrophilic was sample L, made of raw cotton (not mercerized, bleached and grafted with PLA).

**Table 3:** Changes in contact angle values after PLA grafting and active layer implementation.

Sample	Pretreatment	Polymerization	Contact angle after polymerization (with standard deviation) (°)	Contact angle after IBU implementation (with standard deviation) (°)
A	Washing, mercerization (no tension, 20 min)	Bulk (vacuum)	130 (± 0.4)	131 (± 0.3)
B	Washing, mercerization (no tension, 20 min), bleaching	Bulk (vacuum)	112 (± 5.1)	115 (± 2.3)
C	Washing, mercerization (no tension, 3 min),	Bulk (no vacuum)	–	91 (± 7.0)
D	Washing, mercerization (no tension, 20 min),	Bulk (no vacuum)	–	99 (± 3.1)
E	Washing, mercerization (no tension, 3 min)	Bulk (no vacuum)	85 (± 0.7)	90 (± 0.4)
F	Washing, mercerization (no tension, 20 min)	Bulk (no vacuum)	–	91 (± 5.3)
G	Washing, mercerization (no tension, 20 min)	Solution	125 (± 0.8)	125 (± 0.1)
H	Washing, mercerization (under tension, 3 min)	Bulk (no vacuum)	92 (± 6.1)	95 (± 1.1)
I	Washing, mercerization (under tension, 20 min)	Bulk (no vacuum)	94 (± 1.5)	94 (± 1.1)
J	Washing, mercerization (under tension, 3 min)	Bulk (no vacuum)	100 (± 0.8)	98 (± 0.9)
K	Washing, mercerization (under tension, 20 min)	Bulk (no vacuum)	103 (± 0.2)	102 (± 0.7)
L	Washing	No	Not applicable	–
Raw (reference sample)	Washing	No	–	–

All these results show the importance of not only the method of grafting polylactide on cotton but also its pretreatment, which influences the properties of the polylactide obtained in the grafting step of dressing preparation.

### Ibuprofen release profile

**Figure 3:**
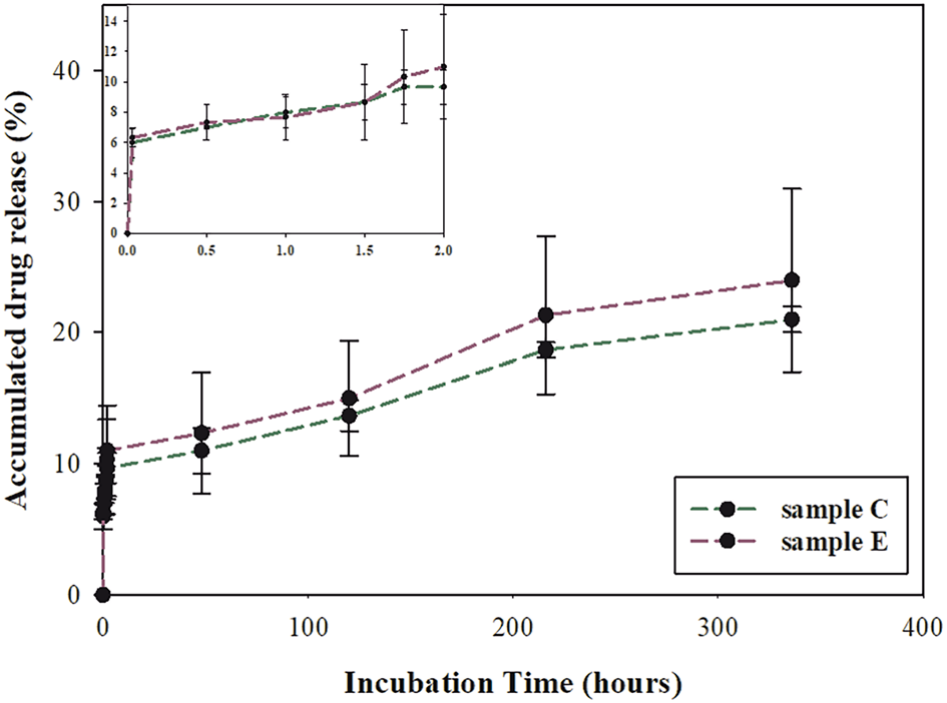
IBU release profile for C and E samples.

Two samples were chosen for this part of the examination, marked with codes C and E. Both were prepared identically, including entirely cotton base pretreatment, polymerization, and active layer implementation. The only difference was the applied drug carrier, which, for sample C, was PLA-PEG, and star-PLA for sample E. This allowed for a reliable comparison of both carriers in terms of drug release rate and a quantitative comparison of ibuprofen released in PBS solution. The experiment was conducted for up to 14 days (Table 4). It was assumed that the maximum amount of attached ibuprofen could not exceed 1 mg/cm^2^; therefore, the content percentage is given in relation to this value. All data was compared to the calibration curve. Initially, a rapid release of the drug into the medium was observed. After two min, 64 μg of ibuprofen was detected, and after 120 min, the amount of the drug increased approximately 1.8-fold to a value of 97 μg (from the PLA-PEG sample C) and 106 μg for the star-PLA sample E (Fig. 3). This phenomenon is known in the literature as burst release, meaning the rapid and often unfavorable release of the drug into the external environment^51^. Wang et al. reports that the burst effect is often caused by high drug content, insufficient binding of the drug to the matrix, diffusion of the drug onto the surface during the manufacturing processes, and higher surface area over volume in the material^52^. In this case, all these points are possible reasons for the initial ibuprofen profile. First, 1 mg of drug per square centimeter of the sample, which is twice the amount of a commercial dressing, was envisaged to saturate the modified cotton fabric. Some researchers showed greater efficiency in covering cotton materials with microcapsules when the bath in the exhausting method is oversaturated with the active ingredient^29^; however, it is associated with its enhanced loss in wastewater. Secondly, a woven fabric itself has expanded physical structure that allows the components of the bath to penetrate and bind them chemically and mechanically by entrapping. Also, the shape of the samples (which were flat fabrics) favored the surface bonding of ibuprofen. Due to the specific application conditions of the experiment, which combine the properties of cotton fibers and fabric’s modifications, considering identical preparation conditions, it can be understandable that in the first stage of drug release, both carriers behave essentially the same, which may be explained by the greater influence of the structure of the entire dressing than the properties of the top active layer. However, further observations of ibuprofen release showed a slight advantage of the star-PLA, which released higher amount of IBU (30 μg) after two weeks than PLA-PEG. At the end of the measurements period, the amount of the released drug reached 209 μg/g for sample C, and 239 μg/g for sample E, which is respectively 21 and 24% of the theoretical drug loading. It is worth mentioning that after the initial burst, the kinetics line was rising smoothly, and it can be cautiously predicted that the advantage of star-PLA in releasing the amount of IBU would have increased over time. However, the difference between those composition is not relevant; therefore, both samples fulfil the requirement for the prolonged anti-inflammatory action.

**Table 4:** Percentage and absolute values of IBU release in 14 days with standard deviation (SD).

Time	Average drug release for sample C and SD (%)	Average drug release for sample E and SD (%)	Average drug release for sample C and SD (μg)	Average drug release for sample E and SD (μg)
2 min	6 (± 0.98)	6 (± 0.05)	63 (± 5.97)	64 (± 0.54)
30 min	7 (± 0.39)	7 (± 0.08)	69 (± 2.38)	72 (± 0.76)
60 min	8 (± 0.56)	8 (± 0.14)	80 (± 3.44)	77 (± 1.37)
90 min	9 (± 0.89)	9 (± 0.50)	87 (± 5.43)	86 (± 4.95)
105 min	10 (± 1.19)	10 (± 0.41)	97 (± 7.26)	103 (± 4.05)
120 min	10 (± 1.24)	11 (± 0.04)	97 (± 7.60)	106 (± 0.39)
48 h	11 (± 1.34)	12 (± 1.52)	111 (± 8.24)	123 (± 1.57)
5 days	14 (± 1.08)	15 (± 0.85	135 (± 6.64)	151 (± 5.66)
9 days	19 (± 0.55)	21 (± 1.16)	188 (± 3.36)	214 (± 11.57)
14 days	21 (± 0.57)	24 (± 2.10)	209 (± 3.49)	239 (± 20.98)

### Biocompatibility of mouse and human fibroblasts exposed to the tested dressings

**Figure 4:**
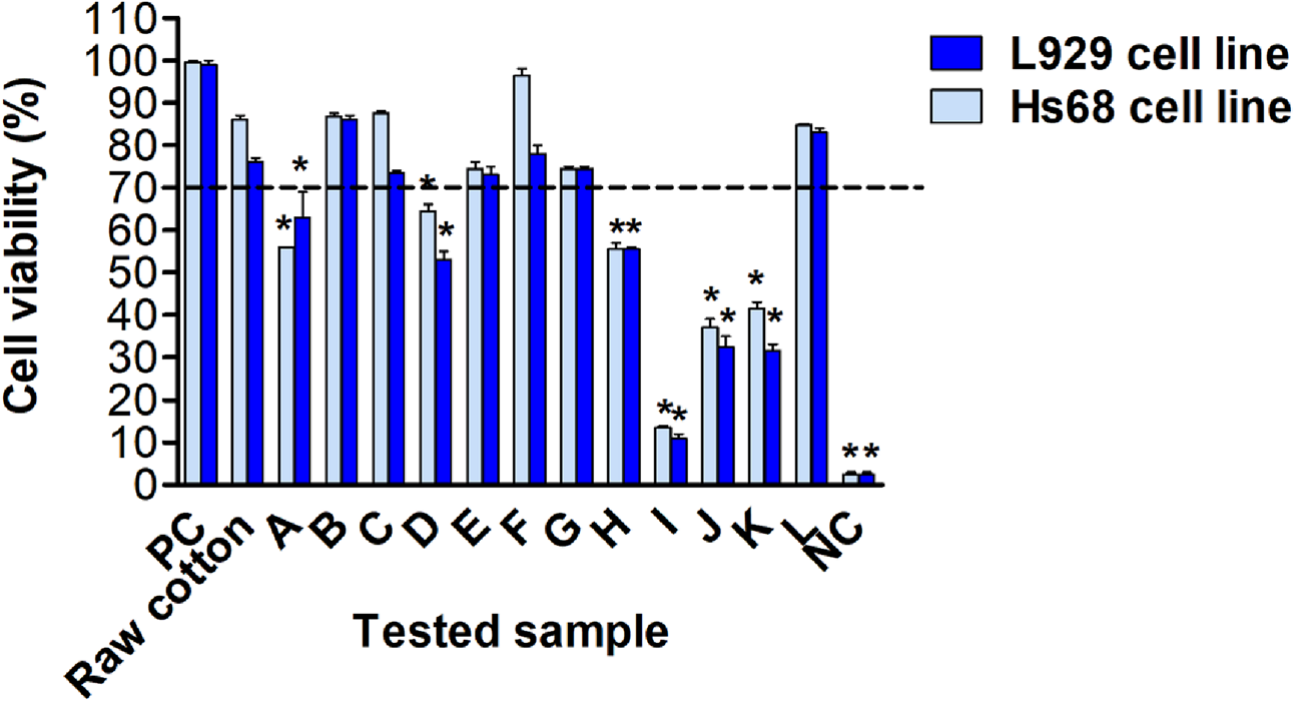
The influence of all tested materials on cell viability in MTT reduction assay. The tested materials: raw cotton, A, B, C, D, E, F, G, H, I, J, K and L were added to L929 fibroblasts or human Hs68 fibroblasts cell cultures. The cell viability was estimated as a percentage of cells, which were able to reduce tetrazolium salt (3-(4,5-dimethylthiazol-2-yl)-2,5-diphenyltetrazolium bromide) (MTT). *NC* negative control (cells treated with 0.03% H_2_O_2_), *PC* positive control (cells in medium alone), raw cotton—tested materials before IBU application. Results are showed as mean ± SD. The black line indicates the minimal percentage of viable cells (70%) required to confirm the biomaterial as non-cytotoxic at the in vitro level. Three experiments were performed in triplicates for each experimental variant. Statistical analysis was performed using the nonparametric U Mann–Whitney test with significance, p < 0.05 (asterisk: unstimulated cells vs. stimulated cells).

Biomaterials for medical applications destined for implantation or contact with tissues for a long time have had to meet particular in vitro cytocompatibility criteria, even in the early stages of composite optimization (ISO 10993-5:2009). In this study, in addition to standard L929 mouse fibroblasts, which are recommended by ISO standards for biocompatibility assessment of tested materials, row cotton and A-L samples were also examined using human Hs68 fibroblasts. The results on the safety of the biomaterials are presented in Fig. 4. The influence of all tested materials on the viability of the reference L929 mouse fibroblasts and Hs68 fibroblasts was assessed by MTT reduction assay based on the measurement of mitochondrial dehydrogenase activity in the presence or absence of the tested substances. The color intensity of dissolved formazan crystals corresponds to the metabolic activity of tested fibroblasts.

The acquired results show that the viability of L929 and Hs68 cells incubated in the presence of tested raw cotton, as well as B, C, E, F, G, and L samples alone, was higher than 70%, meeting the biological safety standard (Fig. 4). Other testing materials: A, D, H, I, J, and K do not meet the biocompatibility standard (viability of both types of fibroblasts below 70%). Regarding the sample preparation method, this group includes all samples mercerized under tension (H, I, J, K), the sample mercerized and polymerized in mass in vacuum (A), and the sample from prolonged (20 min) mercerization (D). These cases imply the importance of the conditions for carrying out the mercerization step. Mercerization conducted in more stringent conditions resulted in greater availability of hydroxyl groups acting as the polymerization initiator. Subsequently, bulk polymerization in a vacuum, which allows producing a high-molecular weight PLA, presumably resulted in incorporation of higher amount of ibuprofen. Thus, it can be cautiously hypothesized that the cytotoxicity is due to ibuprofen itself, although some reports confirm its safety in nonwovens up to a concentration of 1 mg/mL^53^. However, the initial burst of the drug can be an issue, and the properties of the skin barrier, through which IBU permeates due to various biological aspects^54^.

### Anti-inflammatory activity

**Figure 5:**
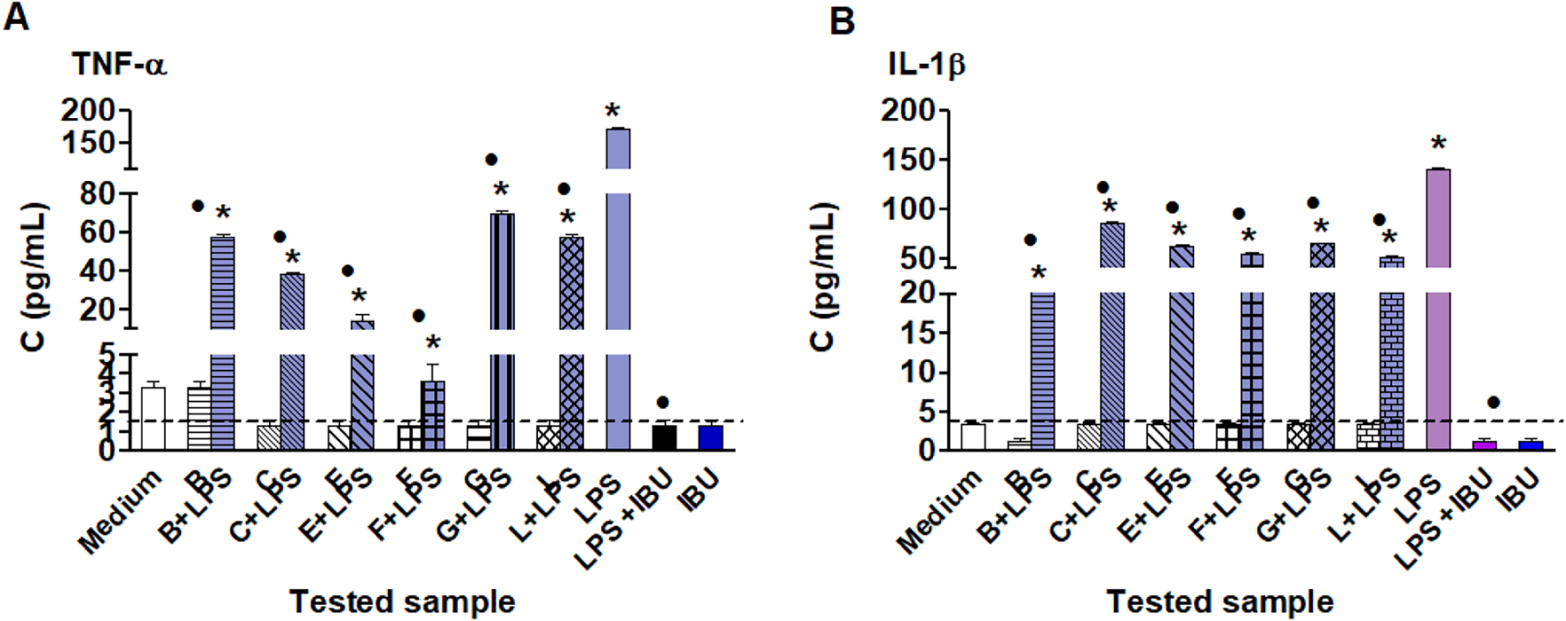
Production of pro-inflammatory TNF-a (**A**) and IL-B (**B**) by human Hs68 fibroblasts. The human Hs68 fibroblasts were stimulated with LPS from *E. coli* alone, as well as with samples B, C, E, F, G and L alone or samples in combination with LPS from *E. coli* (1 µg/mL) or with IBU alone and with LPS from *E. coli* in combination with ibuprofen (IBU. 50 µg/mL). Control cells were subcultured in the medium alone. The supernatants were collected for the assessment of interleukin (IL)-1β or IL-TNF-a by the enzyme-linked immunosorbent assay (ELISA). The results are presented as mean values ± SD. Three experiments were conducted in triplicates for each experimental variant. Statistical analysis was performed using the nonparametric U Mann–Whitney test with significance, p < 0.05 (asterisk: unstimulated cells vs. stimulated cells); or Kruskal–Wallis test (filled circle: LPS vs LPS + dressings).

The occurrence of chronic wounds is increasing with an aging population in the Western world. Patients with chronic wound**s** often experience pain during dressing changes^55^, and the removal of the dressing itself can be associated with an inflammatory reaction. The reasons why chronic wounds fail to heal are several and interrelated—wounds are often poorly vascularized and hypoxic, commonly inflamed, often infected, and consequently aggressive and resistant to healing. That's why we wanted to determine if the cotton dressing with a layer based on biodegradable PLA with ibuprofen may act as an anti-inflammatory agent. Ibuprofen can be used to reduce pain for the patients and reduce the excessive inflammation of the wound bed, which can prevent healing. In our study, we examined the anti-inflammatory potential of the tested materials which met the criterion of biocompatibility: B, C, E, F, G, and L in in vitro models of human fibroblasts Hs68, as well as excluded cytotoxic activity on this line. We chose fibroblasts to check the anti-inflammatory activity of the tested biomaterials study because they are essential cells during the inflammatory, proliferative and maturation phases of normal wound healing, being involved in the formation of several extracellular matrix components, including collagen^56,57^. Results show that the production of pro-inflammatory IL-1β and TNF-a cytokines by human fibroblast induced by LPS from *E. coli* was significantly reduced in the milieu of the B, C, E, F, G, and L, depending on the tested biomaterial. Samples B, C, E, F, G, and L did not stimulate HS68 fibroblasts to produce TNF-a (Fig. 5A) or IL-1B (Fig. 5B). These cells treated with *E. coli* LPS produced TNF-a at a concentration of 175 pg/mL and IL-1B at a concentration of 150 pg/mL. The production of both cytokines driven by LPS *E. coli* was inhibited by ibuprofen and significantly reduced in the milieu of samples B, C, E, F, G, and L. The best anti-inflammatory properties showed sample B (in relation to IL-1B) and F (in relation to TNF-a). These results demonstrate the ability of the tested samples B, C, E, F, G, and L to negatively modulate the production of pro-inflammatory cytokines TNF-a and L-1B fibroblasts treated with LPS *E. coli.*

## Conclusions

While new ways of non-oral delivery of NSAIDs are still being explored, a dressing acting against pain and inflammation, with flexibility achieved from fabric structure and ease of use, was designed. In this paper, various techniques were examined to achieve an active material based on cotton fabric with anti-inflammatory properties obtained through the implementation of IBU in PLA or PLA-PEG layer. The experiment included the initial preparation of the cotton fabric, its compatibilization with polylactide drug carriers, application of ibuprofen and confirmation of its performance over time. Six out of 12 versions differing in terms of modification conditions and the type of ibuprofen carrier decreased the production of cytokines TNF-a and L-1B fibroblasts treated with LPS from *E. coli.* The remaining samples showed cytotoxicity, which were probably caused by a high dose of ibuprofen. Since, in each case, the theoretical amount of drug applied to the dressing was constant and unchanging (1 g/cm^2^), this leads to the conclusion that the primary cause of cytotoxicity was the modification of the material carried out in a way that favored excessive IBU entrapping. The results suggest that drug attachment is influenced by a combination of mercerization, bleaching and polylactide grafting, and the more stringent conditions imposed on individual steps of preparation of dressing (such as mercerization under stress, vacuum polymerization, omission of the bleaching), the greater the likelihood of cytotoxicity.

To sum up, this study does not exhaust the topic of cotton dressings, especially in terms of the precise conditions defined for their preparation, as there is still room for research on avoiding the side effects of implementing the drug on the skin in such a way. However, there are some arguments that they have the potential to be developed as prolonged analgesic and anti-inflammatory materials for wound healing or transdermal drug delivery.

## Data Availability

The datasets generated during and analyzed during the current study are available from the corresponding author on reasonable request.
